# The third delay: understanding waiting time for obstetric referrals at a large regional hospital in Ghana

**DOI:** 10.1186/s12884-017-1407-4

**Published:** 2017-07-11

**Authors:** David M. Goodman, Emmanuel K. Srofenyoh, Adeyemi J. Olufolabi, Sung Min Kim, Medge D. Owen

**Affiliations:** 1 Department of Obstetrics & Gynecology, Duke University Medical Center, Box 3084, Durham, NC 27710 USA; 2 Ridge Regional Hospital, Accra, Ghana; 3 Department of Anesthesiology, Duke University Medical Center, Box 3094, Durham, NC 27710 USA; 4 Wake Forest School of Medicine, Medical Center Boulevard, Winston-Salem, NC 27157 USA; 5 Department of Anesthesiology, Wake Forest School of Medicine, Medical Center Boulevard, Winston-Salem, NC 27157-1009 USA

**Keywords:** Low-middle income countries, Obstetric triage, Obstetric referral, Ridge regional hospital, Ghana

## Abstract

**Background:**

Delay in receiving care significantly contributes to maternal morbidity and mortality. Much has been studied about reducing delays prior to arrival to referral facilities, but the delays incurred upon arrival to the hospital have not been described in many low- and middle-income countries.

**Methods:**

We report on the obstetric referral process at Ridge Regional Hospital, Accra, Ghana, the largest referral hospital in the Ghana Health System. This study uses data from a prospectively-collected cohort of 1082 women presenting with pregnancy complications over a 10-week period. To characterize which factors lead to delays in receiving care, we analyzed wait times based on reason for referral, time and day of arrival, and concurrent volume of patients in the triage area.

**Results:**

The findings show that 108 facilities refer patients to Ridge Regional Hospital, and 52 facilities account for 90.5% of all transfers. The most common reason for referral was fetal-pelvic size disproportion (24.3%) followed by hypertensive disorders of pregnancy (9.8%) and prior uterine scar (9.1%). The median arrival-to-evaluation (wait) time was 40 min (IQR 15–100); 206 (22%) of women were evaluated within 10 min of arrival. Factors associated with longer wait times include presenting during the night shift, being in latent labour, and having a non-time-sensitive risk factor. The median time to be evaluated was 32 min (12–80) for women with hypertensive disorders of pregnancy and 37 min (10–66) for women with obstetric hemorrhage. In addition, the wait time for women in the second stage of labour was 30 min (12–79).

**Conclusions:**

Reducing delay upon arrival is imperative to improve the care at high-volume comprehensive emergency obstetric centers. Although women with time-sensitive risk factors such as hypertension, bleeding, fever, and second stage of labour were seen more quickly than the baseline population, all groups failed to be evaluated within the international standard of 10 min. This study emphasizes the need to improve hospital systems so that space and personnel are available to access high-risk pregnancy transfers rapidly.

## Background

As the global health community works to achieve the Sustainable Development Goals (SDG), it is evident that reducing the worldwide maternal mortality ratio to <70/100,000 live births will require significantly improved systems of healthcare delivery [1]. In low-income countries, obstetric care is focused on providing skilled care for home births and encouraging institutional delivery at community and district hospitals [2, 3]. However, 15% of women will develop complications such as obstructed labour, hypertensive disorders of pregnancy (HDoP), or obstetric hemorrhage (OH) that require transfer to a tertiary level of care capable of performing the signal functions of comprehensive emergency obstetric care (CEmOC) [4]. The inevitable need for escalating care introduces delay into the system and, for many years now, delay has been recognized as one of the root causes of maternal deaths [5, 6].

Much has been written about reducing delays in deciding for referral and reaching referral sites, but less attention has been given to reducing delays once a woman has reached tertiary care [6]. The necessity of frequently receiving high-acuity patients led to the development of obstetric triage as a function of high-quality labour wards. Obstetric triage is defined as “the brief, thorough, and systematic maternal and fetal assessment performed when a pregnant woman presents for care, to determine priority for full evaluation” [7]. This function is most frequently performed by nurses and nurse-midwives. It is more thorough than the type of triage performed in trauma situations as it includes periods of monitoring for labour evaluation, fetal well-being, and laboratory assessment of obstetric complications.

Over the last 30 years, the practice of obstetric triage has been implemented throughout the United States and other high-income countries. In many obstetric units in these hospitals, there is a separate triage area with dedicated staff to receive and rapidly assess women in order to quickly treat complications [8]. The Association of Women’s Health Obstetric and Neonatal Nurses (AWHONN) recommends that the triage assessment begin within 10 min of arrival to a facility [7]. The goal for triage is to conclude the evaluation with a disposition so that the woman can either be discharged home safely or continue with inpatient care. Understanding referral reasons and triage practices is critical for improving maternal health in the new SDG era.

From 2007 to 2011, obstetric admissions increased from 6049 to 9357 at Ridge Regional Hospital (RRH) in Accra, Ghana, a major obstetric referral center for the Ghana Health Service (GHS). An initial pilot survey (data not included) and an analysis of care processes identified bottleneck areas within the labour ward and a decision was taken to study referrals and timeliness of care upon patient arrival [9]. This study characterizes obstetric referrals received at RRH and analyzes the timeliness through which women enter CEmOC.

## Methods

RRH in Accra, Ghana was selected as the site for this study as the highest volume obstetric unit of 10 regional referral hospitals in the GHS. Regional hospitals primarily manage complicated pregnancies and as such, approximately 70% of deliveries at RRH are high-risk antenatal or peripartum referrals. The maternity unit at RRH has a 90-bed capacity and provides comprehensive services from antenatal care through postpartum discharge. In 2012, there were 10 labour and delivery beds, one obstetric operating room, and four general operating rooms shared among surgical services and located remotely from the labour ward. The obstetric triage area was an open hallway with a bench and a small adjacent examination room. Staffing consisted of only two obstetricians, an average of four medical officers/residents, and 22 midwives to manage the operating room and labour ward. Despite these challenges, the unit maintained an open-door policy of not turning away patients needing maternity care. Morning shifts were conducted from 0800 to 1400, afternoon shifts from 1400 to 2000 and night shifts from 2000 to 0800, during which there were typically 4 midwives scheduled during the day shifts and 3 midwives during the night shifts.

Prior to this study, we conducted a small pilot survey among patients that identified waiting time as a significant modifiable factor that negatively affected patient experience and outcome [9]. We developed a data collection and analysis plan to further understand this issue. The a priori goal of the study was to document the wait time and triage time for women when they arrive. We also wanted to identify factors that led to prolonged delays so that an educational and systems-based intervention could be developed. Four non-staff nurses were hired and trained to collect data on obstetric patients admitted to RRH during a 10-week period from September 9 to November 11, 2012. This sample time represented a time of the year with intermediate patient volume based on monthly census data and was selected to reduce the potential influence of peak or low volume periods. Data collectors were scheduled to work throughout the day and night to gather time-sequence information at patient arrival and from patient records and logbooks within 24 h. Data included patient and labour characteristics, referral information, and the timeliness of triage. Timeliness was based on direct observation of patient-provider interactions by the data collection nurses and recorded on a data sheet. We defined wait time as the difference in minutes from arrival at the facility to the first interaction with a midwife. Triage time was defined as the time from first interaction with a midwife to departure from the triage area en route to a treatment area (women’s ward, labour ward, operating theatre, etc.).

### Data analysis

For variables that were normally distributed, Student’s t-test and one-way ANOVA was used for continuous variable, and Pearson chi-squared test was used for categorical variables. Results are shown with means and 95% confidence intervals (CI) where applicable. For variables, such as wait time, that are nonparametric, more appropriate tests were chosen. The Wilcox rank-sum (also known as Mann-Whitney U) test was used for continuous variables and Krukal-Wallis test for categorical variables. These results are reported using medians and interquartile ranges. Statistical analyses were done using STATA version 14.0 software (StataCorp, College Station, TX).

## Results

Over a 10-week period from September 9 to November 11, 2012, data were captured for 1082 women who presented to RRH as transfers from other facilities or self-referrals. This represents 80% of the 1351 deliveries at RRH that occurred during this period. Twenty percent of women were not captured due to the following reasons: admitted directly from clinic, thus bypassing triage; admitted prior to study period, but delivering during the 10-week window; presenting during lapses in data collection nurse coverage. There were 108 sites that referred patients to RRH during the data collection period. There was a wide array of referring facilities ranging from private maternity homes to academic medical centers. The most distant referral sites were 50 km from RRH, a trip that would likely require several hours to complete depending on the time of day. Half of the referrals to RRH came from 9 facilities and the remaining half came from 99 other facilities.

Table 1 shows maternal and labour characteristics upon admission for this population. There were notable gaps in compliance in recording maternal vital signs and in labour assessment. Most notably, maternal temperature was poorly recorded, as well as the presence or absence of uterine contractions. Table 2 shows the reasons for referral as provided by the referring institution. The most common reason for referral was fetal-pelvic disproportion. In a subset of these, 90 patients were referred for prolonged first or second stage of labour, yet 41 arrived with intact membranes. Also, the local vernacular “big abdomen” or “big baby” was used in 90 of these referrals. Twenty-five of these were potentially inappropriate referrals because the fundal height was <40 cm, which would not support this diagnosis. Of the 139 patients referred for hypertension, 13 had normal blood pressure at the time of admission. Two-hundred (18%) of referred women came in advanced labour (>7 cm cervical dilation) and of those, 83 (8%) arrived completely dilated.

**Table 1:** Maternal and obstetric characteristics

Variable	Number (%) observed	Mean	S.D.	Min	Max
Maternal age (yr)	1066 (99)	28.1	5.7	15	46
Maternal heart rate	702 (65)				
Systolic blood pressure (mmHg)	950 (88)	123	25.3	0	220
Diastolic blood pressure (mmHg)	950 (88)	77	16.2	0	140
Temperature (°C)	791 (73)	36.6	0.94	30	40.5
Gravidy	1047 (97)	2.6	1.6	1	13
Parity	1040 (96)	1.4	1.5	0	8
Gestational age (wk)	1000 (93)	39 + 1	24.7	24 + 0	49 + 0
Fundal Height (cm)	954 (88)	37.1	3.72	22	57
Uterine contractions	118 (11)				
Cervical examination (cm)	941 (87)	4.1	2.6	0	10
Fetal heart rate (beats/min)	912 (84)				
Membrane status	926 (86)				
Presentation	1017 (94)

**Table 2:** Indications for referral

Indication	Number	Percent (%)
Fetal-pelvic size disproportion^a^	346	24.3
Hypertensive disorders of pregnancy^b^	139	9.8
Prior uterine scar^c^	129	9.1
Maternal miscellaneous^d^	115	8.1
Anemia^e^	103	7.2
Self-referral/Ridge Hospital patient	92	6.5
Fetal compromise^f^	69	4.8
Fetal malpresentation^g^	62	4.4
Rupture of membranes^h^	54	3.8
Labour	45	3.2
Lack of resources at referral site^i^	43	3.0
Infectious causes^j^	39	2.7
Acute haemorrhage^k^	39	2.7
Prematurity^l^	29	2.0
Previous poor obstetric outcome^m^	27	1.9
Multiple gestation^n^	26	1.8
Record illegible	22	1.5
Maternal age extremes (≤15 or >35 yr)	18	1.3
Intra-uterine fetal demise	14	1.0
No/poor prenatal care	12	0.8
Fetal miscellaneous^o^	2	0.1
Total	1425	100%
One referral indication	739	68.3%
Two referral indications	315	29.1%
Three referral indications	28	2.6%

There were 1082 referral records captured for deliveries occurring at Ridge Regional Hospital from September 9, 2012 to November 11, 2012

^a^Cephalopelvic disproportion, fetal macrosomia, large maternal abdomen, post-term pregnancy, over 40 weeks estimated gestational age, borderline pelvis, contracted pelvis, failure to progress (delayed or prolonged labour, arrest of labour, slow progress, failed induction, unfavorable cervix, high head in labour, obstructed labour)

^b^Chronic hypertension, PIH, pre-eclampsia, severe pre-eclampsia, or eclampsia

^c^Previous cesarean delivery, prior myomectomy, or previous uterine rupture

^d^Maternal asthma, diabetes, gestational diabetes, prior abdominal surgery, uterine fibroids, vaginal/vulvar growth or discharge, proteinuria, urinary tract infection, fever, generalized edema, short/long pregnancy interval, short maternal stature, maternal distress, sterilization request, grand multiparty, seizure disorder, mental illness, obesity, patient refusal for care, patient lacks labouratory or scan information, crippled, rhesus negative

^e^Maternal anemia or sickle cell disease

^f^Abnormal cardiotocography, fetal tachycardia, fetal distress, oligohydramnios, meconium stained amniotic fluid, decreased fetal movement, intrauterine growth restricition, umbilical cord prolapse

^g^Face/mentoposterior, brow, breech/footling breech, oblique, transverse, unstable lie, arm prolapse, leading twin breech, compound presentation

^h^Rupture of membranes, prolonged rupture of membranes, loosing liquor, gestations >37 weeks

^i^No electricity, no bed, no gloves, no water, no doctor, no anesthetist

^j^Hepatitis B, malaria, syphilis, human immunodeficiency virus

^k^Placenta previa, placental abruption, placenta accreta, ante-, intra- and postpartum bleeding, unclassified haemorrhage

^l^Gestation <37 weeks, prematurity, preterm labour or preterm premature rupture of membranes

^m^Bad obstetric history, prior stillbirth, prior ectopic pregnancy, unexplained history of intrauterine fetal death, previous failure to progress, prior cervical cerclage, previous peripartum haemorrhage

^n^Twin pregnancy, triplet pregnancy

^o^Anencephaly, severe hydrocephalus, polyhydramnios, fetal deformity

For women presenting to RRH, the median wait time from arrival until initial assessment by a labour ward midwife was 40 min (interquartile range 15–100 min) (Table 3). Two-hundred and six (22%) women were evaluated within 10 min of arrival, and 41% percent of women were evaluated within 30 min. Seven percent of women were not evaluated within at least 3 h and two women waited longer than a day. A doctor was consulted for 288 (27%) of patients (consultant 42, medical officer 151, house officer 93, 2 unknown). Only 62% of women had a plan of care documented in the chart.

**Table 3:** Wait time and triage time analysis

Factor	N (%)	Wait time (minutes)	IQR (minutes)	*p*-value	N (%)	Triage time (minutes)	IQR (minutes)	*p*-value
Shift								
Morning	325 (32%)	37	11–84	0.0001	253 (28%)	41	13–200	0.0945
Evening	306 (30%)	30	12–84		352 (40%)	40	13–200	
Night	390 (38%)	55	15–135		282 (32%)	70	15–293	
Daily Volume								
< 10 pts	80 (9%)	50	19–92	0.2304	85 (9%)	45	20–380	0.0285
10–19 pts	554 (60%)	40	15–102		555 (60%)	44	15–250	
20–29 pts	210 (23%)	32	10–85		201 (22%)	33	11–143	
> 30 pts	81 (9%)	37	5–125		86 (9%)	83	10–361	
Day of the Week								
Sunday	130 (14%)	47	18–107	0.3767	121 (14%)	70	20–365	0.2316
Monday	122 (13%)	40	15–110		114 (13%)	40	15–213	
Tuesday	118 (13%)	36	12–100		113 (13%)	55	15–310	
Wednesday	129 (14%)	35	12–91		124 (14%)	43	15–231	
Thursday	147 (16%)	40	15–70		142 (16%)	33	10–235	
Friday	146 (16%)	39	10–95		144 (16%)	38	10–174	
Saturday	134 (14%)	44	12–120		130 (15%)	60	16–208	
Weekday	662 (72%)	40	14–192	0.1253	637 (72%)	40	12–227	0.0071
Weekend	263 (28%)	45	15–111		251 (28%)	65	20–298	
Risk Factor								
Other	529 (57%)	45	15–110	0.0299	486 (55%)	60	15–310	0.0079
Sepsis	65 (7%)	35	10–70		68 (8%)	51	18–182	
OH	44 (5%)	37	10–66		42 (5%)	24	12–185	
HDoP	285 (31%)	32	12–80		283 (32%)	34	11–171	
Labour Status								
Latent	489 (56%)	44	15–108	0.0279	476 (56%)	85	20–336	0.0001
1st Stage	275 (31%)	35	10–83		271 (32%)	24	10–65	
2nd Stage	42 (5%)	30	12–79	Ob	45 (5%)	10	5–32	
Not labouring	69 (8%)	38	15–90		51 (6%)	60	15–365	

### Factors associated with wait times and triage times

**Fig. 1:**
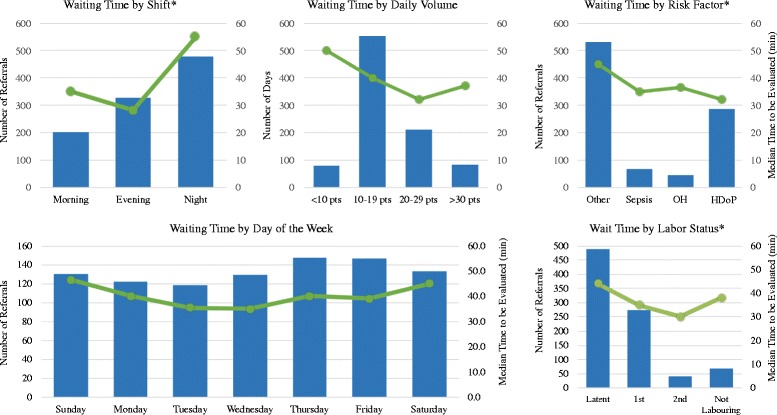
Association of wait time with other variables of interest

We hypothesized that several factors might correlate with faster initial evaluation. We evaluated the time differences from arrival to initial assessment as the “wait” time, and the time from initial assessment to transition beyond the triage assessment as “triage” time. We compared performance around these metrics with respect to the following: time of day, day of the week, volume on a given day, presence of risk factors, and labour status (Table 3, Fig. 1). Arrival times were evenly distributed according to number of hours/shift during the morning (25%), evening (22%), and night (53%) shifts. The median wait time for evaluation was significantly longer at night [55 min (15–120)], than was the morning [35 min (10–83) and evening [28 min (12–51)] shifts (*P* = 0.0004) (Table 3). There was no difference based on day of the week either in volume or wait times (*P* = 0.38).

The impact of volume on wait and triage times showed a non-linear relationship. With respect to wait time, no difference was noted (*P* = 0.23) between groups stratified in groups of 10 patients/day. Moving women out of the triage area took significantly longer on high-volume days (>30 patients) when the median triage time was 83 min (*P* = 0.0285), possibly the result of occupied labor beds. The three most common causes of maternal death at RRH have been shown to be OH, HDoP, and sepsis [10]; thus, we identified women presenting with vaginal bleeding (*n* = 39), hypertension (*n* = 139), fever (*n* = 1), or prolonged rupture of membranes (*n* = 54) as having a time-sensitive risk factor. Women with these risk factors were seen more quickly, 35 min (12–80 min) compared to 45 min (15–110 min) (*P* = 0.009) for women without these risk factors (Table 3, Fig. 1).

Being in labour, either 1st stage or 2nd stage, was associated with being evaluated and being moved out of triage into the labour ward more quickly. Women in labour were evaluated within 35 min (10–83 min) and 30 min (12–79 min) (*P* = 0.0279) for the 1st and 2nd stages respectively, and moved out of triage within 24 (10–65 min) and 10 min (5–32 min) (*P* = 0.0001) respectively (Table 3, Fig. 1).

## Discussion

The results of this study show that regional hospitals face significant challenges receiving, evaluating, and treating many high-risk obstetric referrals. To the best of our knowledge, we report the first large-scale evaluation of delays incurred with obstetric triage in a low-to-middle income country. The analysis presented is intended to describe referral characteristics and delays that occur while receiving patients at a high volume obstetric referral hospital and to inform the development of context specific obstetric triage and staffing strategies to overcome challenges.

The leading indications for referral to RRH were failure to progress (24%) and HDoP (10%). These were similar to the analysis by Nkyekyer et al. who found that the primary reason for referral to Korle Bu Teaching Hospital (KBTH), the main academic medical centre in Accra, was failure to progress (22%) and hypertensive disorders (16%) [11]. Referral for prior uterine scar was only seen in 8 (2%) of patients in the Nkyekyer study; however, this was the third leading indication for patients who presented to RRH constituting 9% of referrals. The increase in these referrals is concerning because it may indicate a rising cesarean delivery rate in Accra over the last few years. Many of these patients were referred from institutions without operating theatres and should have been identified and referred earlier in pregnancy and prior to labor. A continued rise will ultimately lead to an increase in the cesarean delivery burden on regional and large referral hospitals [12].

The Greater Accra Region (GAR) has 17 districts and municipalities for which RRH is responsible. Within GAR there are 4 polyclinics, 31 health centers, and 38 community health and planning services that provide care to pregnant women within the public sector [13]. There are a host of other private and district-level institutions. Two other hospitals, 37 Military Hospital and KBTH, are capable of providing CEmOC and are located within the catchment area. The longest distance traveled by our patient population was 50 km, which was incurred prior to wait time and triage time. Inappropriate and unnecessary referrals also overburden referral hospitals and may contribute to delay in attending to more critically ill patients. Although we didn’t specifically examine accuracy of the referring diagnosis, from the patient folders we found that 41 of 90 patients referred for prolonged labor had intact membranes; 25 of 90 parturients with “big baby” diagnosis had fundal height < 40 cm, and 13 of 139 with diagnosed hypertension had normal blood pressure on arrival. Our analysis indicates that further study and planning is required to optimize the referral patterns and indications and presents an opportunity to add structure to the referral process within the city.

Eighteen years ago, Nkyekyer et al. found that 27% of women reached KBTH by ambulance, whereas 59% relied on taxis for referral [11]. Interestingly, this is consistent with our preliminary 2010 pilot survey in which most patients reported arriving by taxi [9]. This is concerning because the present study found that 200 patients arrived at RRH in advanced labour, 93 of whom were completely dilated. The prospect is frightening of high-risk, pregnant women in advanced labour being transported across the city in taxis and other non-medical vehicles to reach the referral hospital. This is another area where more information is needed in order to improve the referral processes in Accra.

In this study, the volume of patients ranged from 5 to 38 patients/day and there were no differences in volume based on day of the week. Wait times were similar each day of the week; however, it was more likely for patients to wait longer for assessment at night—during shifts with lower staffing. Our analysis shows that an equal number of patients present overnight as do during the daytime shifts. Nursing managers and administrators should make provisions for this observation in order to prevent delays from occurring during overnight. It is reassuring that having a time-sensitive reason for referral does increase the likelihood for quicker evaluation, but this does not reach the AWHONN goal of 10 min, or a more feasible 30-min goal between arrival and evaluation, which was the policy of the hospital.

Our study shows that there was at least a trend towards improved performance with respect to wait and triage times on days with moderately-high volume (20–29 patients) compared with low-volume or the high-volume days. It may be that days of moderately-high volume effectively activate the staff to move through the triage process more quickly. Based on these outcomes we hypothesized that the system should be modified to ensure a reduction of wait and triage times and this will be the focus of future reports.

Studying the outcomes immediately following obstetric referral in Accra can provide guidance to other major cities in Sub-Saharan Africa. Accra has a skilled antenatal care rate of 96% and skilled delivery coverage that ranges from 79 to 84% [13]. These are reassuring achievements and are building blocks for the country to reach their SDGs. As these goals are reached, hospitals will inevitably see an increase in referrals. The early work presented in this study can serve as baseline for planners and a comparison for future efforts.

## Conclusions

Our study shows that RRH is capable of receiving and caring for large numbers of obstetric referrals on a daily basis. We demonstrated that they have a large number of referring facilities, some of which are remotely located, that present a significant burden to women and contributes to delay in their care. Although the median wait time prior to evaluation was 40 min, we believe training and systems improvement could enable the staff to reach a local goal of 30-min evaluation for all patients. Further research is needed in this area in order to establish triage as an integral part of the package of CEmOC in low-resource settings.

## Abbreviations

AWHONN: Association of women’s health obstetric and neonatal nurses
CEmOC: Comprehensive emergency obstetric care
GAR: Greater Accra Region
GHS: Ghana Health Service
HDoP: Hypertensive disorders of pregnancy
KBTH: Korle Bu Teaching Hospital
OH: Obstetric haemorrhage
RRH: Ridge regional hospital
SDG: Sustainable development goals
